# CD4^+^CD25^+^ cells in multiple myeloma related renal impairment

**DOI:** 10.1038/srep16565

**Published:** 2015-11-13

**Authors:** Hongdong Huang, Yang Luo, Yumei Liang, Xi-Dai Long, Youming Peng, Zhihua Liu, Xiaojun Wen, Meng Jia, Ru Tian, Chengli Bai, Cui Li, Xiaoqun Dong

**Affiliations:** 1 Division of Nephrology, Beijing Shijitan Hospital, Capital Medical University, P.R. China; 2 Division of Nephrology, Hunan Normal University, Hunan Provincial People’s Hospital of China, Changsha, P.R. China; 3 Department of Liver Surgery, the Affiliated Renji Hospital, Shanghai Jiao Tong University School of Medicine, Shanghai, P.R. China; 4 Hunan Key Laboratory of Nephrology and Hemoperfusion, Division of Nephrology, Second Xiangya Hospital of Central South University, Changsha, Hunan Province, P.R. China; 5 Section of Hematology/Oncology, Section of Gastroenterology, Stephenson Cancer Center, Department of Internal Medicine, College of Medicine, The University of Oklahoma Health Sciences Center, USA

## Abstract

CD4^+^CD25^+^ cells are critical regulators in almost all of the animal models of human organ-specific autoimmune diseases, transplant rejection and allergic diseases. We aimed to explore the role of CD4^+^CD25^+^ cells in the pathogenesis of multiple myeloma (MM) related renal impairment (RI). Thirty patients with MM related RI and 30 healthy volunteers were studied. The number of CD4^+^CD25^+^ cells was examined by flow cytometry. Clinical and laboratory data were collected from each subject. Glomerular injury was assessed by histopathology. Serum IL-2, IL-4 and IL-6 were analyzed by ELISA. CD4^+^CD25^+^ cells significantly decreased in MM related RI patients compared to the controls (*P*<0.05). CD4^+^CD25^+^ cell number was negatively associated with blood urea nitrogen (BUN), supernatant IL-4, serum IL-6, monoclonal immunoglobulin and β2-microglobulin, as well as bone marrow plasma cell percentage and proteinuria; whereas positively associated with estimated glomerular filtration rate (eGFR) (all *P* < 0.05). CD4^+^CD25^+^ cells gradually decreased as the Clinic Stage increased. The number of CD4^+^CD25^+^ cells reduced in MM related RI patients, and was correlated with disease severity. CD4^+^CD25^+^ cells may play an important role in the pathogenesis of MM related RI.

Multiple myeloma (MM) is a cancer of plasma cells characterized by accumulation of abnormal plasma cells in the bone marrow, presence of serum and/or urinary monoclonal protein (paraprotein, an abnormal antibody causing kidney disease), lytic bone lesions^1^,^2^,^3^, anemia, hypercalcemia, or renal failure. Multiple myeloma is usually diagnosed with serum protein electrophoresis, serum free κ/λ light chain assay, bone marrow examination, urine protein electrophoresis, and X-rays of commonly involved bones. Renal impairment (RI) is a common complication of systemic MM and it impacts on patients’ overall survival. Up to 50% of newly diagnosed patients have a decrease in creatinine clearance (a good estimation of the glomerular filtration rate [GFR]) and ~9% requires dialysis due to severe renal insufficiency^4^. Despite some progress in polychemotherapy regimens with improvements in response rates, the median survival time of those patients remains no more than 2–3 years^5^. High-dose chemotherapy (HDT) supported by autologous bone marrow or peripheral blood stem-cell transplantation has achieved higher complete remission (CR) rates and prolonged event-free and overall survival^6^. Unfortunately, patients with renal failure are often excluded from aggressive or high-dose chemotherapy protocols because of an expected higher toxicity rate. Up to now, the pathogenesis of MM-RI is still unclear. Recent studies have shown that CD4^+^CD25^+^ regulatory T cells are of great importance to the maintenance of tumor-induced immune tolerance by inhibiting the activation and proliferation of autoreactive T cells^7^. CD4^+^CD25^+^ regulatory T cell depletion is indicated to sensitize an established tumor to immunotherapy. Depletion of the minor CD4^+^CD25^+^ cells results in the development of organ-specific autoimmunity^8^. Autoimmune diseases can be prevented by reconstitution with CD4^+^CD25^+^ cells in animal models^8^. Powrie *et al*.^9^ demonstrated that transfer of CD4^+^CD25^+^ cells protected mice from the development of inflammatory bowel disease and even reversed established gastrointestinal inflammation. It has been well accepted that CD4^+^CD25^+^ cells are key regulators of human organ-specific autoimmune diseases, transplant rejection and allergic diseases^10^.

To date, there are only few studies of CD4^+^CD25^+^ cells in human MM related RI. Some patients do not suffer from this disease, implying the possibility to establish a balance between the immunity and tolerance. However, many patients do suffer severally from MM related RI. We therefore hypothesize that a numerical and/or functional deficit of CD4^+^CD25^+^ cells may trigger the development of MM related RI.

## Methods

### Subjects

Thirty patients (14 men and 16 women; age range 41–65 years; mean age 54.8 ± 9.74 years) with MM related RI were enrolled. All the patients were hospitalized in Hunan Provincial People’s Hospital and Second Xiangya Hospital of Central South University between January 2009 and February 2014. Multiple myeloma was diagnosed with bone marrow plasma cells ≥15%, presence of serum and/or urinary monoclonal protein (except in patients with true non-secretory MM), plus evidence of lytic bone lesions^1^, anemia, hypercalcemia, or renal failure. RI patients with MM had renal damages and/or functional impairment. Patients with monoclonal gammopathy of undetermined significance (MGUS)^2^, smoldering multiple myeloma (SMM)^2^, primary amyloidosis^11^, Waldenström’s macroglobulinemia^12^,^13^,^14^,^15^,^16^, and solitary plasmacytoma^17^ were excluded from the study. Thirty healthy volunteers (13 men and 17 women; age range 43–64 years; mean age 58.2 ± 6.75 years) were randomly selected as the control groups in this study.

This study was approved by the Ethical Committee of Hunan Provincial People’s Hospital and Second Xiangya Hospital of Central South University. Informed consent was acquired from each participate before the operation. Each subject had signed written informed consent. All the experiments were carried out in accordance with the approved guidelines and regulations.

### Calculation of data

Renal function was assessed by estimated glomerular filtration rate (eGFR), which was calculated using CKD-EPI (Chronic Kidney Disease Epidemiology Collaboration) creatinine equation^18^. eGFR = 141 × min(Scr/κ,1)^α^ × max(Scr/κ,1)^−1.209^ × 0.993^Age^ × 1.018 [if female], where Scr is serum creatinine (mg/dL), κ is 0.7 for females and 0.9 for males, α is –0.329 for females and –0.411 for males, min indicates the minimum of Scr/κ or 1, and max indicates the maximum of Scr/κ or 1.

### Collection of Clinical data and samples

After acquiring informed consent, clinical data, peripheral blood and urine samples were collected from each subject. All patients received no treatment with steroid, immunosuppressive agents, angiotensin-converting enzyme (ACE) inhibitor, or AT1 receptor blockers before clinical samples were collected. The laboratory examinations before treatments included urinalysis, complete blood count, serum chemistry, and complement components C3.

### Flow cytometry analysis of CD4^+^CD25^+^ cells

Peripheral blood were mixed and incubated for 30 min at room temperature with 10 μl monoclonal Cy5-labeled anti-human CD3 (Jingmei Biotech), FITC-anti-CD4, and PE-anti-CD25. After 30 minutes incubation, the samples were fixed with 1% paraformaldehyde and analyzed by flow cytometry (Coulter EpicsXL, System2 software; Beckman-Coulter) at Key Laboratory of Nephrology, Second Xiangya Hospital of Central South University. The analysis was restricted to lymphocytes (each sample was examined in replicates).

### Isolation and culture of peripheral blood mononuclear cells (PBMCs)

PBMCs were isolated from heparinized peripheral blood by density gradient centrifugation, using Lymphocyte Separation Medium (Flow Labs, McLean, VA). Cells recovered at the interface were resuspended in RPMI1640 supplemented with penicillin (100 U/ml), streptomycin (100 pg/ml), glutamine (2 mM) and 10% heat-inactivated fetal calf serum (FCS), at a concentration of 3 × 10^6^ cells/ml. Duplicate cultures, with phytohaemagglutinin (PHA; Sigma, St Louis, MO) at 20 ug/ml, were maintained for 24 h at 37 °C in a 5% CO_2_ atmosphere. Cell-free supernatant was obtained by centrifugation at 800 g for 10 min and frozen at −70 °C until assayed.

### Enzyme-linked immunosorbent assay (ELISA)

The serum concentrations of IL-2 and IL-6, and the cell-free supernatant concentration of IL-4 were measured using ELISA kits (R&D Systems, USA) according to the manufacturer’s instruction. All the assays were done in triplicates.

### Renal histopathology examination

Patients at high risk of developing kidney biopsy-related complications were excluded. Finally, renal biopsy was performed in 19 patients with MM-RI. All specimens were subjected to fluorescent and light microscopic examination. For light microscopy, biopsy samples were fixed in 10% buffered formalin, dehydrated, and embedded in paraffin by conventional techniques. Sections were stained with hematoxylin and eosin (H&E), and periodic acid-schiff (PAS). IgG, IgA, IgM, complement component C3, activity indices (AI) and chronicity indices (CI), as well as semi-quantitative renal histology at diagnosis, were evaluated. AI included glomerular hypercellularity, leucocyte exudation, fibrinoid necrosis, cellular crescents, hyaline deposits and interstitial inflammation. CI included glomerular sclerosis, fibrous crescents, renal tubular atrophy and interstitial fibrosis. These alterations were blindly graded semi-quantitatively on a 1+ to 3+ scale (mild, moderate, or marked)^19^. We evaluated relationship between the clinical stage (according to the International Staging System Criteria)^20^ and the renal pathological lesions.

### Immunofluorescence staining for λ and κ light chains

The specimen obtained by biopsy was tested with antibodies against κ and λ free light chains. In the original biopsies, fresh frozen renal tissue was sectioned at 3 μm thickness and air-dried. Sections were washed with phosphate-buffered saline (PBS) for 5 min and then stained with fluorescein isothiocyanate (FITC)-conjugated goat anti-human κ and λ light chain antibodies (ICN/Cappel, Aurora, OH), at 1:100 dilution in 5% bovine serum albumin (BSA). The biopsy sections were fixed in 3.7% paraformaldehyde/PBS at room temperature for 20 min, and then washed in PBS for 3 times. After blocking for 30 min in 5% BSA at room temperature, the sections were incubated with rabbit anti-human κ or λ light chain (DAKO, Carpinteria, CA) at 1:100 dilution in 5% BSA for 2 h at room temperature, and then washed in PBS for 3 times. The sections were incubated with tetramethylrhodamine isothiocyanate conjugated anti-rabbit IgG (Roche Diagnostic Corp., Indianapolis, IN) at 1:100 dilution in 5% BSA for 1 h at room temperature, and then washed in PBS for 3 times. The sections were cover-slipped using aqueous mounting medium. Control slides containing renal biopsies of the patients with amyloidosis that were previously positive for κ and λ light chains were stained in parallel with renal biopsies included in the current study.

### Statistical analysis

Data were examined for normality of distribution using the Kolmogorov-Smirnov test and expressed as mean ± standard deviation (SD) or median and interquartile range. Comparison between the two groups was conducted by independent-sample t-test or non-parametric Mann-Whitney U-test as appropriate. Relationships between different values were examined using Spearman’s correlation tests. A *p*-value of <0.05 was considered statistically significant. All statistical analyses were performed using Graph Pad Prism 5.0 (Graph Pad Inc., USA) and Statistical Package for Social Sciences version 16.0 (SPSS Inc., USA).

## Results

### Flow cytometry analysis of CD4^+^CD25^+^ cells

**Figure 1:**
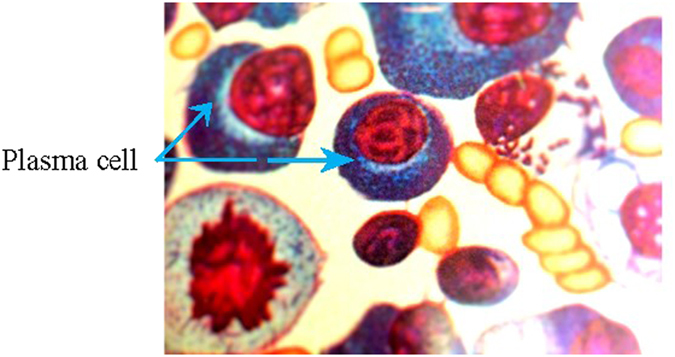
Bone marrow of multiple myeloma (40×).

**Figure 2:**
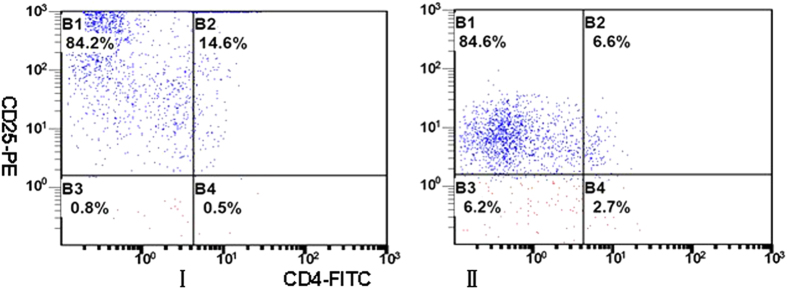
CD4^+^CD25^+^ cells in peripheral blood by flow cytometry. CD4^+^CD25^+^ cells were counted using the indicated gates and enumerated in Table 1. B4: CD4^+^CD25^+^ cells, I: MM-RI patients, II: Control. CD4^+^CD25^+^ cells significantly decreased in MM-RI patients compared to the control (P < 0.05).

A representative image of bone marrow plasma cells in MM patient was shown in Fig. 1. We analyzed the number of CD4^+^CD25^+^ cells in peripheral blood by flow cytometry (Fig. 2). CD4^+^CD25^+^ cells significantly decreased in patients with MM-RI compared to healthy control (^a^*P* < 0.05) (Table 1).

### Clinical and histopathological findings

Clinical characteristics and histopathological findings were listed in Table 2. Serum IL-2, IL-6, and supernatant IL-4, as well as blood monoclonal immunoglobulin and β2-microglobulin significantly increased in patients with MM-RI compared to healthy control (^*^P < 0.05). However, there was no significant difference in serum levels of C3 between MM-RI patients and healthy control (*P* > 0.05).

### CD4^+^CD25^+^ cells and clinical data

The associations between the numbers of CD4^+^CD25^+^ cells and clinical parameters were examined in patients with MM-RI (Table 3). CD4^+^CD25^+^ cells were negatively correlated with serum blood urea nitrogen (BUN), and uric acid, but positively correlated with eGFR (*P* < 0.05). These results indicated that CD4^+^CD25^+^ cells may represent renal function. CD4^+^CD25^+^ cells were negatively correlated with urine protein, supernatant IL-4, serum IL-6, bone marrow plasma cell percentage, as well as blood monoclonal immunoglobulin and β2-microglobulin (*P* < 0.05).

### Correlation between CD_4_^+^CD_25_^+^ cells and Clinic Stage of MM-RI

**Figure 3:**
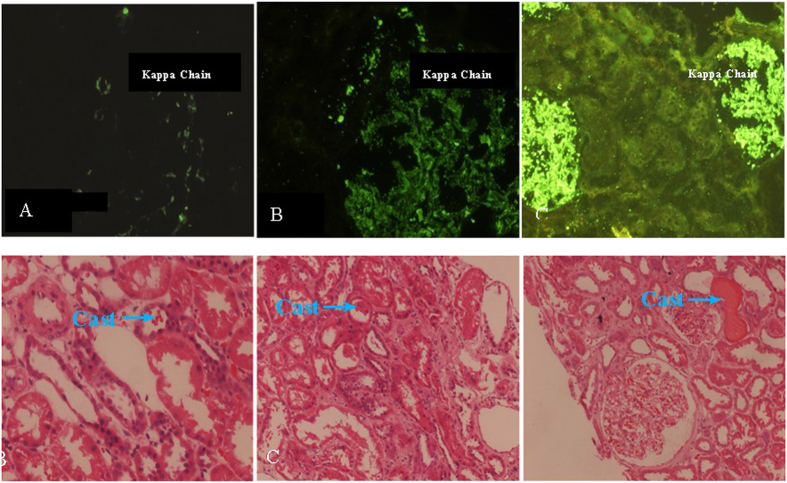
Histopathological findings in MM-RI patients (40×). (**A**) Mild; (**B**) Moderate; and (**C**) Marked lesion.

**Figure 4:**
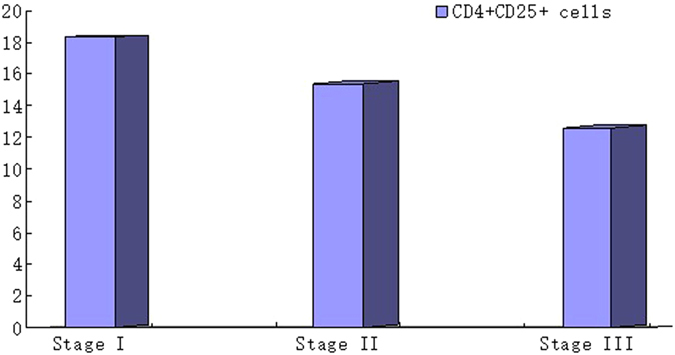
The number of CD4^+^CD25^+^ cells and clinic stages of MM-RI disease. The number of CD4^+^CD25^+^ cells in MM-RI patients tended to decrease in parallel with the Clinic Stage increased, although the difference was not significant (*p* > 0.05).

CD4^+^CD25^+^ cells in MM-RI patients tended to decrease in parallel with advanced clinical stages and severity of renal pathological lesions, although the differences were not significant (*P* > 0.05) (Figs 3 and 4).

## Discussion

Multiple myeloma is a malignant clonal B-cell disorder of slowly proliferating plasma cells, accompanied by monoclonal protein production and lytic bone lesions^21^. Regulatory T lymphocytes (Tregs) are cells that regulate or suppress the function of other immune cells. CD4^+^CD25^+^ cells, a type of Tregs, have been identified in mice and humans as a distinct population of CD4^+^ T cells that constitutively express the interleukin (IL)-2 receptor α-chain (CD25)^22^,^23^. CD4^+^CD25^+^ cells have been shown to dampen local anti-tumor responses and prevent sterilizing immunity against certain chronic infectious agents. On the other hand, CD4^+^CD25^+^ cells occasionally mediate peripheral tolerance, leading to an exacerbated inflammatory/allergic reaction or autoimmunity^24^. Regulatory CD4^+^CD25^+^ T cells are ‘suppressor’ T cells, involved in various diseases^10^. The most notable immunomodulatory property of CD4^+^CD25^+^ cells is their ability to limit the development of a proinflammatory CD4^+^ Th2 phenotype (as demonstrated by reduced cytokine production)^25^. Abnormality of peripheral T cell can result from an in-appropriate balance between allergen activation of CD4^+^CD25^+^ cells and effector Th2 cells^26^,^27^, which is due to a deficiency in CD4^+^CD25^+^ cells or strong activation signals that overcome the suppressor^28^. It has been shown that after antigen inhalation, CD4^+^CD25^+^ cells play a key role in immunomodulation^29^. Th1 (IL-2, IFNγ, and TNF-β) response is more likely to be regulated by CD4+CD25+ cells than Th2 (IL-4, IL-5, IL-6, IL-10, and IL-13) response^30^. Our previous studies^31^,^32^ have shown that tonsillar CD4^+^CD25^+^ Treg cells were significantly decreased in IgA nephropathy as well as thrombotic thrombocytopenic purpura (TTP) associated with systemic lupus erythematosus (SLE). Furthermore, we have found that tonsillar CD4^+^CD25^+^ Treg cells from IgA nephropathy patients present reduced imunosuppressive activity in experimental IgA nephropathy rats^33^. Some patients do not suffer from MM-RI, implying the possibility of a balance between immune activation and tolerance. In contrast, other patients do suffer from MM-RI. We propose that either numerical or functional deficit of CD4^+^CD25^+^ cells may promote the development of MM-RI. In this study, we observed that CD4^+^CD25^+^ cells significantly decreased in MM-RI patients compared to the control. Serum IL-2 and IL-6, supernatant IL-4, urine protein, and urine erythrocytes, as well as blood monoclonal immunoglobulin and β2-microglobulin significantly increased in MM-RI patients compared to the control. CD4^+^CD25^+^ cell numbers were negatively correlated with serum BUN, uric acid, supernatant IL-4, serum IL-6, urinary protein, bone marrow plasma cell percentages, blood monoclonal immunoglobulin and β2-microglobulin, but positively correlated with eGFR in MM-RI patients. CD4^+^CD25^+^ cells in MM-RI patients tended to decrease in parallel with an increase in clinic stages and the severity of renal pathological lesions, although the differences were not significant in those patients. A possible explanation for this observation is that when MM-RI patients experience a decrease in CD4^+^CD25^+^ cells, their lymphocytes react significantly more strongly to antigens, leading to higher levels of cytokine production (IL-2, IL-4, and IL-6). Excessive cytokine production (especially IL-6) may enhance uncontrollable proliferation of plasma cells. Therefore, increased monoclonal immunoglobulin and free light chains are present in the serum of MM-RI patients. It is well-known that free light chains play a crucial role in causing renal damages. Experimental studies in rats and mice have shown that infusion of light chains purified from patients with renal failure can induce tubular cast nephropathy^34^. Cast nephropathy is a typical renal complication found in MM patients. In MM, the capacity of the proximal tubular cells to reabsorb and catabolize light chains is enhanced. Consequently, light chains not reabsorbed in the proximal tubuli reach the distal segment of the nephron where they can combine with the Tamm–Horsfall mucoprotein (THP) to precipitate in the formation of obstructing casts. Obstruction of distal tubuli leads to the leakage of tubular content into the interstitium, and thus contribute to the development of tubular casts and myeloma kidney^21^. In this respect, some light chains that cause Fanconi Syndrome may be digested by cathepsin B. In contrast, other types of light chains causing obstructing casts in the distal tubules are resistant to proteolysis by trypsin and pepsin^35^. Some nephrotoxic light chains appear to be able to self-aggregate into large polymers under physiological conditions in the distal tubuli^36^. Monoclonal immunoglobulin deposit and/or plasma cell infiltration in different regions of kidney can cause renal impairment (such as glomerulonephritis and tubulointerstitial nephritis). Amyloid light chain protein deposit in the glomerular basement membrane, glomerular mesangium or renal tubular basement membrane may lead to renal impairment^37^. Progressive loss of renal function confers a proinflammatory milieu and functional defects in almost all innate and adaptive immune cells. Renal failure can develop rapidly at an unchanged ratio of production and concentration of serum light chains. Various factors can promote renal cast formation in myeloma patients. Dehydration due to diuretics reduces the glomerular filtration rate (GFR) and increases the plasma concentration of light chains, exceeding the capacity of the proximal tubuli for reabsorption and catabolism of light chains. Hypercalcemia may induce vasoconstriction followed by a decrease in GFR. Several drugs, in particular non-steroidal anti-inflammatory agents (NSAIDs), can reduce renal blood flow. Radiographic contrast agents may also induce acute renal failure in myeloma patients, particularly if patients are dehydrated and ionic contrast media are used^34^. Kelley TW *et al*.^38^ observed an increase in CD4+CD25+ cells in multiple myeloma (MM). Our results are different from the mentioned above studies, which may reflect the difference in patient population or severity of MM with RI.

Our findings indicate that CD4^+^CD25^+^ cells may play an important role in the pathogenesis of MM-RI. Altering the CD4^+^CD25^+^ cells number might be useful in the prevention and treatment of MM-RI, which needs further investigation.

Although our findings are both interesting and useful, several limitations should be considered. For example, the relatively small sample size may weaken the statistical power for comparisons. Thus, a comprehensive study on the role of CD4^+^CD25^+^ cells in a large patient population is required to confirm our results. In addition, the functions of CD4^+^CD25^+^ cells *in vivo* should be further explored in various animal models of MM-RI.

In conclusion, the present study suggests that CD4^+^CD25^+^ cells reduce in MM-RI patients, and may correlate with disease severity. CD4^+^CD25^+^ cells may participate in the pathogenesis of MM-RI. Modification of the CD4^+^CD25^+^ cell number might help to prevent and treat MM-RI. Additional studies are necessary to demonstrate the functional deficit and abnormal biological properties of CD4^+^CD25^+^ cells in MM-RI pathogenesis.

## Additional Information

**How to cite this article**: Huang, H. *et al*. CD4^+^CD25^+^ cells in multiple myeloma related renal impairment. *Sci. Rep.* **5**, 16565; doi: 10.1038/srep16565 (2015).

## Figures and Tables

**Table 1:** The number of CD4^+^CD25^+^ cells in peripheral blood.

	MM with RI groups (%)	Controls (%)
CD4^+^CD25^+^ cells (mean ± SD)	0.82 ± 0.106^a^	2.28 ± 0.457

CD4^+^CD25^+^ cells significantly decreased in MM-RI patients compared to the control (^a^*P* < 0.05).

**Table 2:** Clinical and histopathological findings in MM-RI patients and controls.

	MM with RI	Control
SBP (mmHg)	126.8 ± 11.6	124.5 ± 13.5
DBP (mmHg)	77 ± 10.6	75 ± 10.8
Bone marrow plasma cell (%)	43.5 ± 13.2	–
Hemoglobin (g/dL)	7.5 ± 1.1^*^	13.0 ± 1.6
IgG (g/L) (n = 26)	60.19 ± 9.80^*^	12.10 ± 2.61
IgA (g/L) (n = 4 )	38.10 ± 2.72^*^	2.04 ± 0.53
Blood β2-microglobulin (mmol/L)	6.7 ± 1.04^*^	0.91 ± 0.23
sFLC (mg/L)	1876 ± 364^*^	0.963 ± 0.32
Albumin (g/dL)	3.5 ± 0.21^*^	4.1 ± 0.36
Proteinuria (g/day)	3.02 ± 0.15	–
Hematuria (×10^4^ cells/mL)	74.13 ± 6.25	–
BUN (mg/dL)	18.5 ± 2.76^*^	11.34 ± 0.83
Serum Cr (mg/dL)	1.82 ± 0.25^*^	0.74 ± 0.26
Serum uric acid (μmol/L)	441.52 ± 56.08^*^	334.25 ± 63.47
eGFR (mL/min/1.73 m^2^)	80.21 ± 7.83^*^	112.67 ± 7.25
Serum C3 (mg/dL)	106.5 ± 23.91	108.2 ± 26.01
Serum IL-2 (pg/ml)	121.3 ± 10.74^*^	23.4 ± 7.54
supernatant IL-4 (pg/ml)	326.15 ± 73.02^*^	201.36 ± 90.32
Serum IL-6 (pg/ml)	49.56 ± 7.18^*^	27.52 ± 6.82
I/II (ISS)	19	–
III (ISS)	11	–
AI/CI (median)	10.5 ± 2.3/3.4 ± 0.2	–
Kappa (%)	16 (84)	–
Lambda (%)	3 (16)	–

SBP, systolic blood pressure; DBP, diastolic blood pressure; AI, activity index; CI, chronicity index; ^*^P < 0.05 compared to the control.

**Table 3:** Correlation between the proportion of CD4^+^CD25^+^ cells and clinical parameters in MM-RI patients.

	CD4^+^CD25^+^ cells (%)	
	*r*	*p*
SBP	−0.275	n.s.
DBP	−0.189	n.s.
BUN	−0.786	<0.01
Scr	−0.683	<0.01
Sua	−0.598	<0.01
eGFR	0.639	<0.01
24-h UP	−0.587	<0.01
Bone marrow plasma cell (%)	−0.746	<0.01
Serum IgA (n = 4)	−0.715	<0.01
Serum IgG (n = 26)	−0.675	<0.01
Blood β2-microglobulin	−0.639	<0.01
Hematuria	−0.123	n.s.
Serum C3	−0.131	n.s.
Serum IL-2	−0.041	n.s.
Supernatant IL-4	−0.884	<0.01
Serum IL-6	−0.762	<0.01

N.s., not significant; SBP, systolic blood pressure; DBP, diastolic blood pressure; Scr, serum creatinine; Sua, serum uric acid; eGFR, estimated glomerular filtration rate; 24-h UP, 24- hours urinary protein.
